# Complexity and risk factor research in mental disorders: better think twice

**DOI:** 10.3389/fpsyt.2026.1893137

**Published:** 2026-08-21

**Authors:** Vladeta Ajdacic-Gross, Lena Ajdacic, Mario Müller, Ana Buadze, Sebastian Olbrich, Erich Seifritz, Roland von Känel, Martin Preisig, Caroline L. Vandeleur

**Affiliations:** 1 Department of Adult Psychiatry and Psychotherapy, Psychiatric University Clinic Zurich and University of Zurich, Zurich, Switzerland; 2 Social Sciences Institute, University of Lausanne, Lausanne, Switzerland; 3 Department of Consultation-Liaison Psychiatry and Psychosomatic Medicine, University Hospital Zurich, University of Zurich, Zurich, Switzerland; 4 Department of Psychiatry, Psychiatric Epidemiology and Psychopathology Research Center, Lausanne University Hospital and University of Lausanne, Prilly, Switzerland

**Keywords:** complexity, heterogeneity, methodology, neurodevelopmental disorders, risk factors, suicide, systems

## Abstract

Complexity has become an increasingly prominent concept in research on mental disorders and pathological behaviors. This is more than due, as these conditions are not only complicated but genuinely complex. Complexity serves both as an analytic lens and as a force that pushes scientific progress forward. However, complexity science, as it currently stands, seems poised to deliver waves of new findings but no transformative breakthroughs. We propose rethinking complexity from a multisystem perspective which has been common in social sciences and naturally fits with the setting in mental and behavioral disorders, respectively. The key feature of multisystem complexity is heterogeneity of involved systems, heterogeneity of involved processes, their interactions and outcomes. Implementing a multisystem and heterogeneity perspective requires a thorough rethinking of methodology, while assessing failures, misunderstandings, requirements and prospects. The same is true for research directions such as risk factor research. Examples of implementations are drawn from research on suicide and neurodevelopmental disorders.

## Introduction

Biomedical research produces a wealth of new findings, yet advances in understanding occur at markedly different speeds. The research domains considered here – mental and neurodevelopmental disorders and pathological behaviors – tend to belong to the slower category. For a long time, understanding, predicting, and ultimately preventing them seemed to many scholars to be unattainable, even impossible. However, this picture has begun to change. Following the emergence of network concepts and analyses that have signaled a new era in brain research, complexity concepts have also gained increasing attention (1–3) and have been applied across a wide range of conditions and behaviors, including depression (4, 5), suicidal behavior (6), and substance use (7, 8).

At present, the field stands at a crossroad. To advance further – and ultimately to make the seemingly impossible possible – some rethinking is required. “Better think twice” is a deliberately ambiguous suggestion that invites multiple perspectives. It points at both complexity and risk factors as requiring a closer look. At the same time, it suggests that within each domain, rethinking must begin at a fundamental level. In this perspective article, research strategies and methodological tools for addressing complex topics are outlined with examples drawn from research on suicide and neurodevelopmental disorders.

The implications of complexity for epidemiological and risk factor research are difficult to overestimate (9). In many respects, conducting research in this context resembles operating on a different planet.

## Gardeners on the jungle planet – a parable

Imagine a “Green Garden Week” taking place somewhere in space, with a range of events. One of them unfolds as follows: a spaceship carrying gardening enthusiasts lands on a distant, green planet, where new Baroque gardens are to be established. A formidable task – but an even greater obstacle stands in the way. To reach the designated garden area, an obscure, dense, and rugged jungle must first be crossed.

The gardeners immediately unpack their lawn mowers, hedge trimmers, shovels, spades, rakes, shears, and begin forcing their way through the thicket. After a short distance, they return and try again. Each attempt yields new material and information about the forest, but no path forward.

The following day, tools and tactics are changed. Some gardeners turn to high-precision trimmers and cutters; others to big powerful mowers. Some begin to dig and analyze the soil; others climb trees to search for the next clearing. Some few succeed to progress deeper into the jungle, but never come back. Progress improves slightly, yet no reproducible path emerges. Will the gardeners ever reach their destination and begin their sophisticated Baroque work?

Observers of the Green Garden Week broadcast would probably know what is needed to cross the jungle: machetes, axes, chainsaws, and, to maintain an overview, a bunch of drones.

## How is complexity reflected in risk factor research: first impressions

The jungle in the parable can be readily understood as a metaphor for complexity. A high degree of complexity has far-reaching consequences across our research domains – and particularly so in risk factor research (10). A brief look at some key observations illustrates this:

Confirmed or well-supported risk factors already number in the dozens. They appear to be rivaled by genetic markers, which may reach into the hundreds [for schizophrenia, see (11)]. More importantly, they reflect a striking diversity of underlying mechanisms, spanning multiple body and brain systems and subsystems, as well as a wide range of exogenous influences.

At the same time, the wealth of findings does not match the reach of this research: across disorders, diseases, and behaviors, the largest subgroup of affected individuals – depending on the disorder under study: one to two thirds – reports no identifiable risk factors at all.

Beyond their diversity, risk mechanisms and risk factors are characterized by dense networks of interrelations. In this context, disorders and behaviors can be understood as outcomes of interacting processes. One consequence is that research is confronted with equifinality and multifinality: different mechanisms may lead to the same outcome, while similar mechanisms may result in different outcomes. These concepts, originally developed in general systems theory, have been adopted in psychopathology (12) as well as in brain network studies (13) and genetics.

Equi- and multifinality are hallmarks of complex systems. While biomedical research more broadly continues along a path of increasing specialization, risk factor research in psychiatric disorders is confronted with the need to consider not just one system at a time, but many systems in parallel.

## Complexity and multisystem complexity

There are several archetypal examples of complexity:

- bird flocks or fish shoals with their unpredictable formations (multiagent complexity)
- bat-and-ball games such as table tennis, or badminton (multiaction or multifunctional complexity)
- brain networks (network or multilink complexity)
- technical or physiological circuits (cybernetic complexity)
- division of labour and differentiation of societies (multilayer or multisystem complexity)

These examples suggest that complexity is fundamentally about systems – specifically, systems characterized by some form of multiplicity – agents, functions, links, activations, subsystems, or components – and corresponding interconnections.^1^ Attempts to define complexity typically also incorporate characteristic system behaviors and features (see below), yet no definition has proven satisfactory given the diversity of complexity forms and their associated features (14).

Multisystem complexity represents the most comprehensive subtype of complexity, potentially encompassing elements of all others. Importantly, it is particularly well suited to addressing the multifactorial etiology of mental disorders and pathological behaviors. “Systems all the way down” is how Eiko I. Fried and Donald J. Robinaugh characterized this in an insightful formulation (1). Diverse systems – e.g. neural, immune, endocrine, metabolic, structural – interact to maintain function, enable adaptation, and protect against dysfunction.

Formally, most systems are composed of subsystems, forming what has been described as “systems of systems” (François Jacob). This implies that multisystem complexity scales in a hierarchical, potentially exponential manner. Systems and subsystems consist of components, which in turn are composed of further subcomponents, and so on (9, 15, 16). Interactions occur within and between these levels, resulting in multidimensional, multilayered manifestations of complexity.

This scheme is echoed by complex outcomes, for example in the hierarchical relationship between diagnoses and symptoms in mental disorders. While essential for the discipline (17–20), this relationship can be understood as a recurrent “systems of systems” loop: broader categories, such as internalizing and externalizing disorders, comprise diagnoses; diagnoses and their subtypes are defined, among other factors, by symptoms; symptoms can be further subdivided, and these subdivisions can again be subdivided, effectively shifting both benefits and limitations across levels of analysis. Preference for any level is per se arbitrary.

Characteristic features of complex systems are emergence, evolution, adaptation, self-organization, spontaneous order, interference, and chaos. In more technical accounts, emphasis is placed on interactions, nonlinearity, interdependence, feedback loops, and power laws (14). The most important feature within multisystem complexity is heterogeneity – closely intertwined with interdependence, including reciprocity and feedback processes. Notably, heterogeneity has often been overlooked, particularly in approaches rooted in technology and the natural sciences, much like multisystem complexity itself.

At an operational level, heterogeneity is reflected in subtypes or subgroups characterized by specific configurations of features. While subtypes along the vertical axis are defined by configurations of diagnoses and/or symptoms, along the horizontal axis, additional criteria enable further variants of subtyping – for example risk factors, comorbidities, sex, age at onset, disease course, or physiological features. Typically, complex outcomes are characterized by multiple overlapping forms of heterogeneity and, accordingly, by multiple potential subtype configurations that should in principle be considered. Their number may become enormous, even though existing classifications – for example in depression (21) – already appear highly differentiated. While scientific progress often implies increasing differentiation, this development poses substantial challenges for theoretical frameworks, methodologies, and research designs. Suggestions, methodological implications, and two examples—research on suicide and neurodevelopmental disorders—are outlined in the final section of this article.

“All models are wrong, but some are useful” (George E. P. Box): while complexity introduces challenges, it also opens opportunities for more integrative and potentially transformative research. The potential of transdiagnostic frameworks – such as the Research Domain Criteria and the Hierarchical Taxonomy of Psychopathology (22) – depends on adequately addressing multisystem complexity and its key features.

## Multisystem complexity and methodology: something has been going wrong

Undetermined heterogeneity and pervasive interdependence create challenges across many research domains, but their impact on methodology is particularly severe. This becomes evident when considering widely accepted methodological guiding principles:

- clarity (of questions, aims, interpretational framework)
- precision
- parsimony (Occam’s razor)
- noise reduction
- model fit optimization, predictive accuracy
- complexity reduction
- replicability and reproducibility
- reliability
- transparency and accessibility
- transferability/external validity

Under conditions of high complexity, these principles systematically fail to function as intended. For many scholars, the resulting inconsistencies and instabilities come as a surprise. They have been widely discussed, for example in the context of the reproducibility crisis (23). They have fueled debates and criticism across disciplines and between methodologists and applied researchers. However, a substantial part of the reproducibility crisis may simply reflect a complexity-blind science. Apparent deficiencies – such as limited reliability or precision – are not necessarily methodological failures, but properties of the subject matter itself. In this sense, what appears as failure is not at fault, but a default condition of research in complex systems.

A similar pattern can be observed in methods and statistics. Conventional approaches including methods such as meta-analyses (24) are limited in their ability to capture heterogeneity and interdependence in complex systems (25–28). Within a multisystem context, heterogeneity tends to attenuate associations and degrade statistical parameters, while interdependence may inflate them – resulting in a paradoxical or inconsistent pattern of findings. The typical response has been to intensify methodological rigor: increasing precision, tightening thresholds (e.g., adopting p-values of.01 or.001 instead of.05), restricting repeated testing, applying more sophisticated models, expanding datasets, and relying on aggregation methods such as meta-analyses or synthetic approaches such as the use of Artifical Intelligence – much like the gardeners in the jungle parable, who refined their tools yet still failed to find a path through the forest.

Even approaches and analyses that take heterogeneity into account often face substantial limitations. Given that multiple forms of heterogeneity characterize complex outcomes, multiple perspectives and configurations of features must be assessed to achieve a sufficiently comprehensive understanding of the target. Meanwhile, common practice is to focus on a presumably “best” perspective or configuration – whether for pragmatic publication reasons or due to restricted conceptual frameworks. Moreover, analyses often rely on arbitrary combinations of diverse variables originating from different systems and levels, producing results that are difficult or impossible to interpret.

On a more fundamental level, established methodological paradigms – such as experimental designs and Popperian frameworks – offer only limited guidance in the context of high complexity. Similarly, strictly bottom-up approaches tend to produce large numbers of isolated findings without yielding deeper understanding. Addressing multisystem complexity therefore requires a reconsideration and adaptation of methodological principles themselves.

## Rethinking scientific approaches and methodology in the face of complex systems and outcomes

Our research domains – mental disorders and pathological behaviors – do not deal with hypothetical or purely abstract outcomes. Rather, they involve concrete outcomes, despite their heterogeneity, diversity, fuzzy definitions, and dimensional features. They provide an immediate and often uncompromising test of how functional our research actually is – more directly than other fields, such as economics or sociology. Against this background, the following sections outline key directions for advancing the understanding of multisystem complexity, the methodological approach and the outcomes it generates.

### Methodological orientation

In their standard text on artificial intelligence, Stuart Russel and Peter Norvig noted that “to solve a hard problem, you have to almost know the answer already.” (29) p. 40. Although originally referring to expert systems, the same paradox applies to research on complex outcomes. A central requirement is to identify the relevant systems, subsystems, and their interactions, as well as how functions are distributed across them.

To “almost know the answer already,” either theoretical assumptions or substantial prior knowledge are needed. Where such knowledge is lacking, systematic exploratory approaches and analyses focusing on patterns are needed to develop a preliminary overall view on contributing systems and their interactions. In the jungle parable, this corresponds to developing maps with the help of drones.

In both cases, research is essentially oriented top-down, driven by an overall view. Notably, multisystem complexity cannot be assessed by operating bottom-up. Even beyond the well-known features of complex systems – such as emergence, unpredictability, and chaos – the bottom-up approach encounters fundamental limitations in multisystem contexts, which cannot be resolved by increasing data volume and methodological refinement.

Nevertheless, research benefits from iterative processes. The large body of specific findings acquires a new role: it provides a basis for identifying patterns, contributes missing pieces, and helps to challenge or refine the emerging overall understanding.

### Multisystem complexity adapted analytic approaches

At a practical empirical level, the key approach centers on patterns: pattern analysis, pattern recognition, and pattern exploration. Patterns serve to identify heterogeneous outcomes – subtypes and subgroups – as well as the underlying systems and mechanisms. Mechanistically, disentangling subtypes or subgroups transforms high-degree complexity into more manageable levels of complexity, thereby facilitating analysis and interpretation.

In the analysis process, heterogeneity typically comes first, followed by interdependence and other features. The analytical basis can be derived from a wide range of models – simple and sophisticated – which inform about associations. These correspond to the machetes, axes, and saws in the jungle parable. At a more advanced level, clustering models play an important role (21) (e.g., cluster analysis, latent class analysis, multidimensional scaling, factor analysis). Although these models are typically designed to reduce complexity, the key here is to apply them from multiple perspectives and to recombine in a two- or multiple-step procedure the resulting information, i.e. to meaningfully rebuild and reproduce complexity.

In practice, this may involve using different numbers of clusters (analogous to different flight altitudes in the parable), different variable sets representing distinct systems, various statistical models and model combinations. This approach bears some resemblance to multiverse regression analysis (30, 31) and associated models, although it serves a different purpose. In this instance, it is not about assessing the reproducibility of results through controlled model variation, but rather about the diversity of perspectives revealed by different model configurations.

This perspective runs counter to conventional methodological principles such as complexity reduction, parsimony, and model fit optimization. However, some of these principles remain essential at a higher level of abstraction, that is, when applied to patterns rather than individual results (for example, clarity, reproducibility, and transparency). This represents a subtle but important shift. Within a multisystem context, patterns serve as the key lens of research. They provide the appropriate level at which selected methodological principles can be applied and and help to make sense of the multitude of positive and null findings emerging under conditions of heterogeneity and interdependence, including outcomes resulting from poorly conducted research.

Alternatives to pattern-based approaches include dynamical systems modeling (32–34), which focuses on interdependence and temporal dynamics. In most cases, such models are applied to specific targets and remain distant from comprehensive multiscale simulations (13).

Another approach is network analysis, which has gained prominence in the study of brain systems and connectivity. Formally, network models can be applied to any system of associations, such as comorbidities at the diagnostic or symptom level (17, 35). Network analysis offers considerable potential, as it incorporates concepts that extend beyond conventional statistical thinking and is capable of modeling both interdependence and heterogeneity.

### Multisystem complexity adapted scientific approach: strategical lines and beyond

Between methodological orientations and analytic approaches lies a strategic level. The following (incomplete) list outlines key guiding principles:

- Aim to reproduce, not reduce, complexity.
- Adopt a comparative and interdisciplinary approach. The work of Jules Angst (36) provides a notable example. Comparative analyses yield both conceptual perspectives and analytical insights, which are ever more valuable with increasing complexity.
- Embrace the diversity of perspectives rather than focusing on a single “best” one. Introduce the change of perspectives by specific sequences or iterations as a part of an analysis design.
- Accept limited reproducibility and precision as a default condition. Patterns can emerge from both stable and unstable results; the latter are often particularly informative in the presence of contradictions and apparent paradoxes.
- Integrate patterns with gaps, contradictions, and paradoxes. Actively seek out the latter – following, at a smaller scale, principles akin to those described by Thomas Kuhn regarding scientific revolutions (37).
- Work extensively with models. Models serve as a central tool for top-down guidance, structuring analysis and interpretation. It takes several generations of a model to meaningfully orchestrate stable and unstable findings, existing and lacking knowledge.

Beyond guiding priciples, i.e. on the concrete level of research designs and research practice in the context of multisystem complexity, it is clear that a novel and extended operational basis is required both for working with patterns and for continuingly generating specific results. Until this basis will be developed, research would most benefit from a maximum transparency regarding research designs and purposeful model variation. In reporting, this may include not only a full technical reporting of models, but also a discussion of research designs and their implications, for example analogous to the standard limitations sections.

### Risk mechanisms and risk factors

In addressing heterogeneous outcomes, multiple interacting systems typically need to be considered, encompassing several potential risk mechanisms. Beyond this, formal differentiation criteria are needed to reflect the heterogeneity of outcomes. A useful heuristic is to apply a grid of such criteria systematically to risk factor findings. Core criteria include:

- Hierarchical positioning of risk factors within outcome subtypes: necessary and sufficient factors; weak factors with substantial potential (“tip-of-the-iceberg” factors), including proxies for small but theoretically relevant subgroups or subtypes.
- Recurrence of risk factors, recurrence and chronicity of risk mechanisms.
- Occurrence vs. persistence vs. mutual factors: much research does not distinguish between factors operating before and after (biological) onset, limiting insight for example into equifinality and multifinality.
- Proximal risk factors (such as smoking or blood pressure) vs. distal factors (e.g., sex, familial aggregation) and associated factors.
- Risk factors vs. predictors.
- Temporal features I: stages of disease development.
- Temporal features II: age at onset and developmental status of the brain, of physiological and other systems.

### Multisystem complexity-adapted research: two examples

Suicide research provides a first example of a multisystem complexity-adapted approach. Here, comprehensive theoretical and empirical frameworks have existed for more than a century, beginning with Emile Durkheim’s seminal sociological and epidemiological work (38). In our research, we took advantage of this background and pursued two strategies: to address gaps in theoretical concepts and to contribute new findings – dedicated either to filling existing gaps or to exposing new ones.

The gaps in suicide research start at a very basic level with the observation that suicide thoughts and suicides overlap less than commonly assumed. Suicide thoughts are obviously a necessary condition for suicide, indeed a well-studied one in connection with depression and beyond. But given the rates (up to 50% lifetime prevalence of suicide thoughts vs. 1% suicide deaths per generation) this condition is far from sufficient. Another gap arises from the necessary condition of a high suffering load or mental ache, as put forward by psychiatrist and psychologists (39, 40). Again, most of the time high levels of suffering do not result in suicides. These are hypothesized to emerge from impulses shaped by prior mental rehearsal of suicidal scenarios and situational circumstances rather than from conscious planning, as for example in assisted suicides.

In our studies on suicide seasonalities/cyclical effects (41–45), on suicide methods (46, 47), in-patient suicides (46, 47) and other issues (48, 49), we elaborated another necessary condition: opportunities. Opportunities include access to means, acting skills, timing, perceived opportunities (including suicide thoughts), role models, and future perspectives. Conversely, they include protective counterforces such as social contact or control. The overall concept of “opportunity structure” is borrowed from classical criminological research (50). “Structure” in this context means that each suicide rests on a configuration of opportunities, with individual elements becoming essential within a given configuration – while at the same time offering a lever for prevention. Simplifying, suicide may be understood as emerging from the interaction between suffering load, opportunity structures, and situational circumstances.

**Figure 1:**
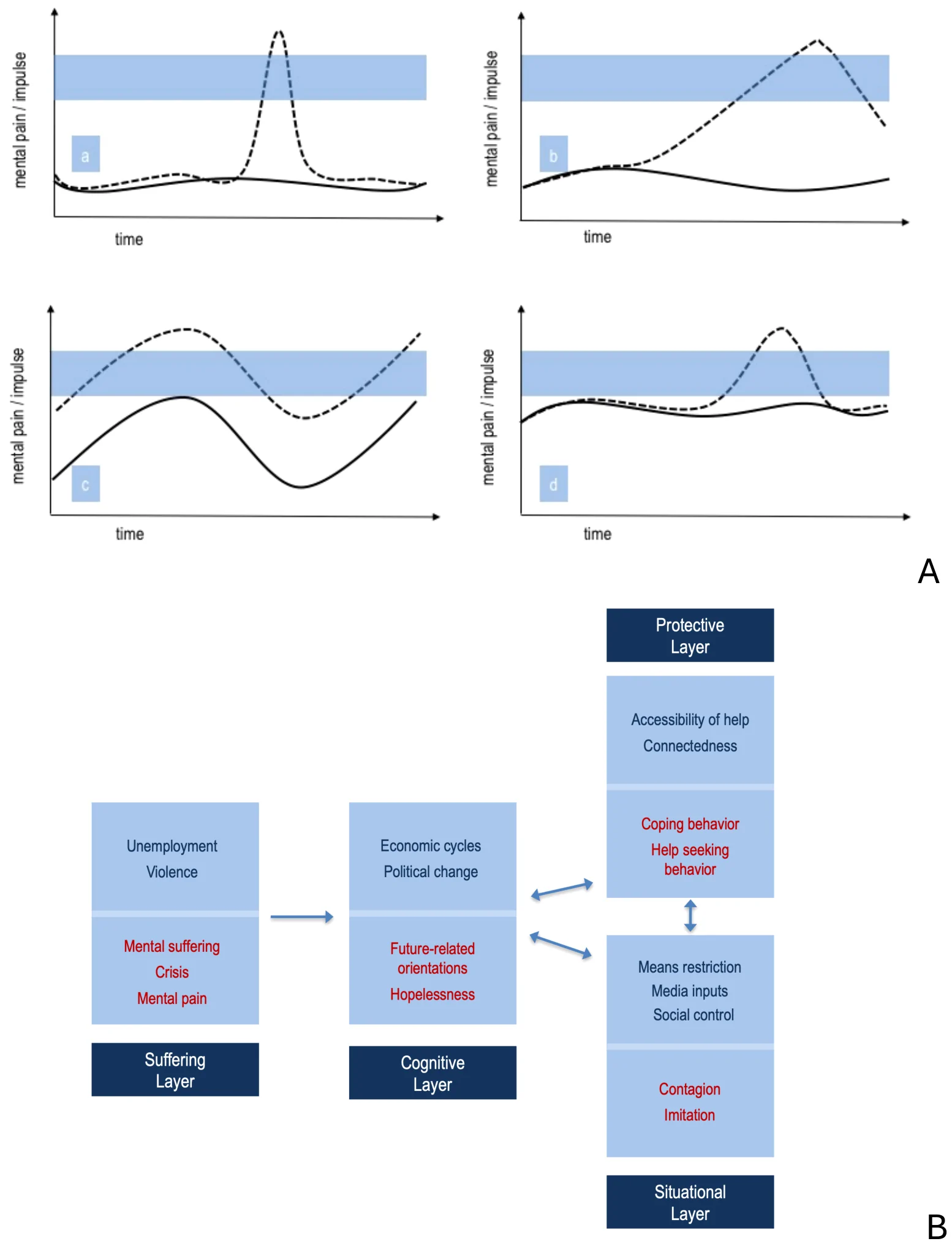
Two complementary interpetational models of suicide. **(A)** Four subtypes of temporal dynamics of suicide impulses: rash act suicides (a), prolonged strain suicides (b), recurring risk suicides (c), balancing act suicides (d). Assisted suicides not shown here. The model components include baseline suicidality (bold line), a deviating course (dotted line), and a hypothetical critical threshold for the start of acting. (Reprinted with permission by Elsevier from:Ajdacic-Gross V, Hepp U, Seifritz E, Bopp (M) Rethinking suicides as mental accidents: Towards a new paradigm. J Affect Disord. 2019 Jun 1;252:141-151. doi: 10.1016/j.jad.2019.04.022). **(B)** Risk mechanism layers in suicide; examples are related to the societal level (upper box part, blue font) and to the individual level (lower box part, red font). (Reprinted with permission by Elsevier from:Ajdacic-Gross V, Hepp U, Seifritz E, Bopp (M) Rethinking suicides as mental accidents: Towards a new paradigm. J Affect Disord. 2019 Jun 1;252:141-151. doi: 10.1016/j.jad.2019.04.022).

This research has been accompanied by a longer history of model development, which found its current double form as shown in **Figures 1a, b**. One model (**Figure 1a**) covers different temporal schemes of suffering, leading to different subtypes of suicides, i.e. suicide heterogeneity. The spectrum extends from “rash act” suicides to assisted suicides, with a huge proportion resembling some kind of accidents – “mental accidents” (51). The second model (**Figure 1b**) represents different layers of opportunity structures stacked upon the suffering layer: cognitive, situational, and protective. Together, the two models provide a comprehensive and deep understanding of suicides. In practice, this framework informed the development of the suicide prevention program in the canton of Zurich, Switzerland (**Figures 1A, B**).^2^

**Figure 2:**
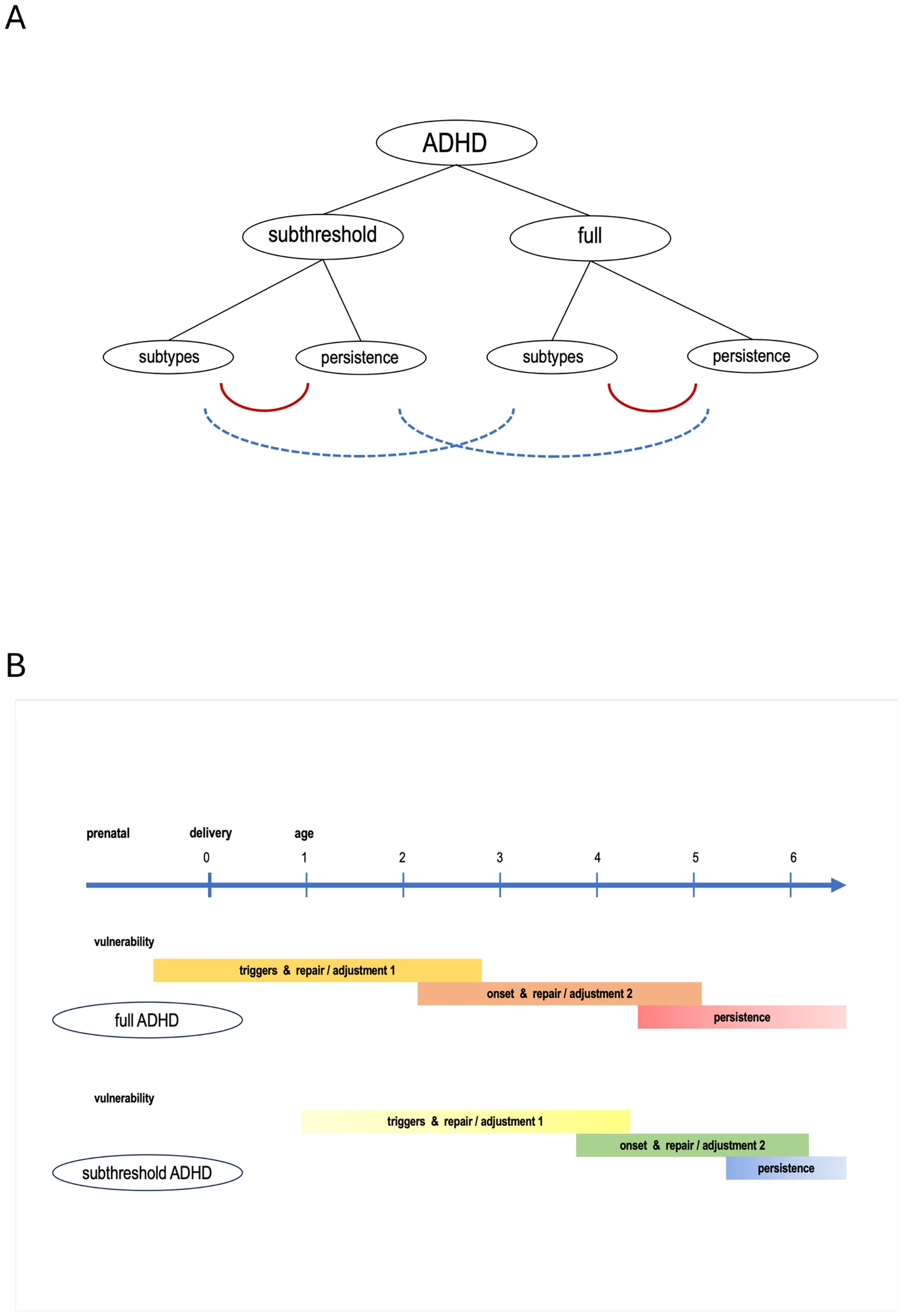
Two models in systematic exploratory research in ADHD: analysis and interpretation. **(A)** Analysis design targeting subthreshold, full and persistent ADHD. (Reprinted based on CC BY license from: Buadze A, Ajdacic-Gross V, Müller M, Xu Y, Seifritz E, Wagner E-YN, et al. (2026) Factors associated with the occurrence and persistence of subthreshold and full attention-deficit hyperactivity disorder in women: A population-based epidemiological study. PLoS One 21(5): e0340179. https://doi.org/10.1371/journal.pone.0340179). **(B)** Timing of risk factors across age stages in full and subthreshold ADHD.

The second example derives from ongoing epidemiological research on neurodevelopmental disorders (ADHD, stuttering, tics) (52–54). In contrast to suicide, the theoretical and empirical resources here are less abundant. We therefore developed a systematic exploratory analysis design to rapidly develop a broader understanding of the multisystem structure and gaps in knowledge. The core of this design is shown in **Figure 2A**. It entails comparisons between full and subthreshold forms, between persistent and non-persistent forms, and identification of subtypes based on risk and associated factors. Beyond this, further comparisons follow across sexes, comorbidity patterns, and, finally, between disorders.

A challenging preliminary insight in ADHD was that risk factors may differ between full and subthreshold forms, and also between occurrence and persistence of a condition (54). Another central finding was a conspicuous difference in age of onset between the full and the subthreshold form in both sexes, with earlier onset in the former and later onset in the latter. Given the rapid pace of brain development in early life, timing matters, and developmental changes may unfold rapidly across short intervals. This is reflected in the interpretational model (**Figure 2B**), which uses an extended vulnerability–stress (or vulnerability–trigger) concept separately for full and subthreshold ADHD. The duplicated and temporally shifted models immediately suggest that risk and protective factors may yield different consequences depending on the brain developmental stage at which they occur. Moreover, they suggest that windows to apply interventions differ accordingly. The model provides a framework for better understanding how risk and pathogenetic mechanisms differ within and between developmental and other early disorders (**Figures 2A, B**).

## Conclusions

In research on mental disorders and pathological behaviors, we are mostly trying to get through dense jungles, rather than planting new Baroque gardens as we intended. Implementing the complexity concept is overdue. In this article we introduce a research framework based on the multisystem complexity concept. This framework has its foundation in systems theory and comprises different levels of methodological implications, from theory of science to the choice of statistical models. Rethinking complexity from a multisystem perspective is essential, with heterogeneity and interdependence as its defining features. This shift has direct implications across multiple domains, including conceptual thinking, revision of methodological guiding principles, design of analytical strategies, and task-adapted use of methods. Disentangling the heterogeneity of pathological processes, risk factors and outcomes is a prerequisite for research on complex psychiatric outcomes that aims for transformative advances. Elements of a systemic perspective are already present in various lines of research, including dynamical systems modeling and network analysis. Making multisystem complexity explicit may help to integrate these directions. Substantial progress will not come from reducing complexity, but from learning how to work with it.

## Data Availability

The original contributions presented in the study are included in the article/supplementary material. Further inquiries can be directed to the corresponding author.

## Footnotes

1 Readers should be careful to distinguish the complex from the merely complicated, and complexity from complicatedness (16, 55, 56).

2 See: https://www.suizidpraevention-zh.ch/suizidpraevention-kanton-zuerich
